# A Disease Identification Algorithm for Medical Crowdfunding Campaigns: Validation Study

**DOI:** 10.2196/32867

**Published:** 2022-06-21

**Authors:** Steven S Doerstling, Dennis Akrobetu, Matthew M Engelhard, Felicia Chen, Peter A Ubel

**Affiliations:** 1 Duke University School of Medicine Duke University Durham, NC United States; 2 Margolis Center for Health Policy Duke University Durham, NC United States; 3 Department of Biostatistics & Bioinformatics Duke University Durham, NC United States; 4 Apple, Inc Cupertino, CA United States; 5 Fuqua School of Business Duke University Durham, NC United States; 6 Sanford School of Public Policy Duke University Durham, NC United States

**Keywords:** crowdfunding, natural language processing, named entity recognition, health care costs, GoFundMe

## Abstract

**Background:**

Web-based crowdfunding has become a popular method to raise money for medical expenses, and there is growing research interest in this topic. However, crowdfunding data are largely composed of unstructured text, thereby posing many challenges for researchers hoping to answer questions about specific medical conditions. Previous studies have used methods that either failed to address major challenges or were poorly scalable to large sample sizes. To enable further research on this emerging funding mechanism in health care, better methods are needed.

**Objective:**

We sought to validate an algorithm for identifying 11 disease categories in web-based medical crowdfunding campaigns. We hypothesized that a disease identification algorithm combining a named entity recognition (NER) model and word search approach could identify disease categories with high precision and accuracy. Such an algorithm would facilitate further research using these data.

**Methods:**

Web scraping was used to collect data on medical crowdfunding campaigns from GoFundMe (GoFundMe Inc). Using pretrained NER and entity resolution models from Spark NLP for Healthcare in combination with targeted keyword searches, we constructed an algorithm to identify conditions in the campaign descriptions, translate conditions to *International Classification of Diseases, 10th Revision, Clinical Modification* (ICD-10-CM) codes, and predict the presence or absence of 11 disease categories in the campaigns. The classification performance of the algorithm was evaluated against 400 manually labeled campaigns.

**Results:**

We collected data on 89,645 crowdfunding campaigns through web scraping. The interrater reliability for detecting the presence of broad disease categories in the campaign descriptions was high (Cohen κ: range 0.69-0.96). The NER and entity resolution models identified 6594 unique (276,020 total) ICD-10-CM codes among all of the crowdfunding campaigns in our sample. Through our word search, we identified 3261 additional campaigns for which a medical condition was not otherwise detected with the NER model. When averaged across all disease categories and weighted by the number of campaigns that mentioned each disease category, the algorithm demonstrated an overall precision of 0.83 (range 0.48-0.97), a recall of 0.77 (range 0.42-0.98), an *F*_1_ score of 0.78 (range 0.56-0.96), and an accuracy of 95% (range 90%-98%).

**Conclusions:**

A disease identification algorithm combining pretrained natural language processing models and ICD-10-CM code–based disease categorization was able to detect 11 disease categories in medical crowdfunding campaigns with high precision and accuracy.

## Introduction

Many patients share details about their health care experiences on the internet. Various platforms, ranging from social media to discussion forums, provide an outlet to convey aspects of the patient experience that may not be captured by feedback surveys or academic studies. For example, researchers have analyzed web-based hospital reviews to understand which hospital quality metrics are the most important to patients [1]. Other work has analyzed Twitter posts to detect adverse drug reactions alongside traditional adverse event reporting systems [2,3]. Considering the public availability and high volume of patient-authored, web-based content, these posts constitute an important data source for gleaning insights about the real-world impact of health care from the patient perspective.

One such data source that has gained recent attention is web-based crowdfunding, which has become a popular method that many in the United States use to raise money for medical expenses. As of April 2019, more than US $3 billion was raised for personal medical expenses on GoFundMe (GoFundMe Inc)—the largest web-based crowdfunding platform. To understand how different patient populations are impacted by medical expenses, recent studies have used data from GoFundMe to identify campaigns associated with specific, narrowly defined medical conditions, focusing on, for example, cancer [4,5], injuries [6], or neurologic diseases [7]. However, because GoFundMe campaigns do not contain any structured data on medical conditions, these details must be inferred from the free text of each campaign description. To address this challenge, a variety of methods have been explored to identify campaigns associated with specific medical conditions, including manual reviews [8]; rule-based approaches based on keywords and regular expressions [9]; and, more recently, biomedical word embeddings for establishing similarities to reference words for broad disease categories [6].

Each of these approaches has important shortcomings. Rule-based approaches might systematically overlook misspelled diagnoses or the conversational phrasing of medical terms. Manual reviews are time intensive and thus scale poorly to larger sample sizes. Strategies based on biomedical word embeddings are promising but are highly context dependent and may perform unpredictably with crowdfunding campaigns because of frequent misspellings and vague medical terminology. Additionally, most medical crowdfunding studies have focused on a single or small number of disease categories, and disease categories are often treated as mutually exclusive at the campaign level [8,10,11], even though many people seek money to pay for the cost of multiple illnesses.

Considering these challenges, better methods are needed to answer important questions about the scale and impact of medical crowdfunding. To facilitate this work, we sought to construct an algorithm to more accurately and comprehensively identify medical diagnoses in medical crowdfunding campaigns. We used a named entity recognition (NER) model, which can be trained to predict phrases that represent medical conditions and have been successfully applied to medical corpora for disease identification [12]. Medical conditions identified by the NER model were then converted to *International Classification of Diseases, 10th Revision, Clinical Modification* (ICD-10-CM) codes to group conditions into disease categories. In this paper, we present data on the precision and reliability of a new algorithm that was designed to detect the presence or absence of 11 mutually inclusive disease categories in medical crowdfunding campaigns.

## Methods

### Data Collection

We wrote a web scraping program to collect data from medical crowdfunding campaigns that are hosted by GoFundMe. The program accessed a random sample of the GoFundMe sitemap [13], which contains links to GoFundMe crowdfunding campaigns that are made available to search engines. Web scraping was completed in August 2020. Data were collected from campaigns that were self-categorized as *Medical, Illness & Healing* and located in the United States.

### Ethics Approval

This study was approved by the Duke University Institutional Review Board (IRB number 2020-0435). All data collected from GoFundMe were publicly available and aggregated for research purposes in accordance with fair use. The source code is available on GitHub [14].

### Disease Identification and Resolution to the ICD-10-CM

In order to identify medical diagnoses in the descriptions of crowdfunding campaigns, we used an NER model developed by Spark NLP for Healthcare [15]. The NER model identifies segments of text that are predicted to represent medical diagnoses. Each text segment that was identified as a medical diagnosis was subsequently entered into an entity resolution model, which was also developed by Spark NLP for Healthcare [16]. The entity resolution model selects the ICD-10-CM codes that most closely match the input text according to the distance between embedding vectors. Together, this pipeline generates a list of medical diagnoses and their corresponding ICD-10-CM codes for each campaign description.

### Categorizing ICD-10-CM Codes

Our goal was to sort ICD-10-CM codes into clinically coherent disease categories. We used the 2021 Clinical Classifications Software Refined (CCSR; Healthcare Cost and Utilization Project) for ICD-10-CM diagnoses [17], which groups ICD-10-CM codes at the following two levels of specificity: a narrow CCSR clinical category (eg, *Heart failure*) and a broad diagnosis chapter (eg, *Diseases of the Circulatory System*). ICD-10-CM codes from certain CCSR clinical categories were reassigned to a different diagnosis chapter to consolidate the number of disease categories and prioritize a system-based classification of diseases (Multimedia Appendix 1). For example, congenital abnormalities were reassigned to categories related to the impacted organ systems. Afterward, we selected 11 diagnosis chapters for disease categories that we sought to identify in crowdfunding campaigns. These categories were chosen because they represented common medical conditions in the United States and were suitable for principal diagnoses that are made according to ICD-10-CM documentation. The diagnosis chapters included for our analysis were renamed to differentiate them from those in the official ICD-10-CM and CCSR documentation (Multimedia Appendix 2). The final assignment of ICD-10-CM codes to disease categories is provided in Multimedia Appendix 3.

### Identification of Disease Categories by Using a Word Search

Our research team, which was comprised of a senior physician, 2 medical students, and research assistants with undergraduate and master’s degrees, conducted several rounds of exploratory reading of crowdfunding campaigns to understand how medical details were conveyed. We observed that crowdfunding campaigns sometimes did not explicitly state a medical diagnosis but instead referenced a procedure or treatment that implied the presence of a diagnosis. For example, mentioning *chemotherapy* suggests the presence of a neoplasm. Considering that the NER model used in our study was trained to identify medical diagnoses and not procedures or treatments, campaigns that failed to mention a diagnosis would be missed. Other pretrained NER models exist for the detection of treatments and procedures, but the use of these models was outside the scope of this project. Instead, we compiled a list of treatments and procedures that appeared during our team’s review and assigned each term to a disease category. If a term was present in a campaign description, we indicated that the term’s corresponding disease category was present in the campaign.

### Recoding ICD-10-CM Codes

**Figure 1:**
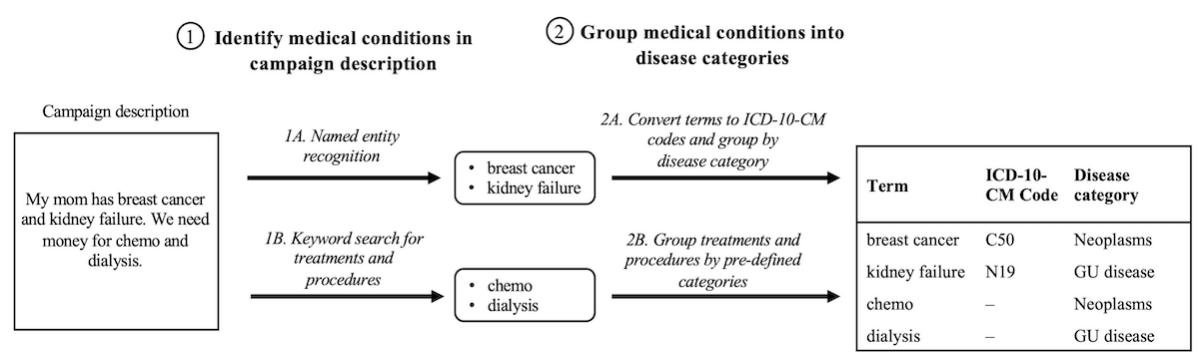
A schematic diagram of the disease identification algorithm. This figure shows how this study’s algorithm determines which disease categories are present in a hypothetical example that is representative of web-based medical crowdfunding text. Medical conditions are identified in the text by using a named entity recognition model to identify diagnoses and keyword searches to identify treatments and procedures. Diagnoses identified by the named entity recognition model are assigned to best-matching ICD-10-CM codes by using an entity resolution model and grouped according to the disease category definitions outlined in the *Methods* section. Treatments and procedures were used to indicate the presence of corresponding disease categories (defined in Table 1). GU: genitourinary; ICD-10-CM: *International Classification of Diseases, 10th Revision, Clinical Modification*.

Certain ICD-10-CM codes that were identified by the entity resolution model did not have an exact match in the CCSR data that were used to group codes into disease categories. To align these codes from the entity resolution model with the CCSR data, we removed the last character of the unmatched code, thereby creating a trimmed code, and checked if any code in the CCSR data began with the resulting trimmed code. This process was repeated until a match was found. If multiple CCSR codes were found to begin with the trimmed code, then the unmatched code was assigned to the disease category that was the most common among the matched CCSR codes. The final set of recoded ICD-10-CM codes was then merged with disease categories that were derived from CCSR, thereby aligning each campaign’s identified medical conditions with their corresponding disease categories. ICD-10-CM codes that mapped to the *Other* category were then removed. Each remaining disease category was summarized as “present” or “absent” for all campaigns. A schematic of the algorithm is shown in Figure 1.

### Evaluation of Classification Performance

We created a manually labeled reference set to evaluate the ability of our algorithm to detect medical diagnoses in crowdfunding campaign text. A subset of campaigns (n=400) was independently reviewed by 2 medical students, which we considered as the ground truth. The reviewers identified medical diagnoses in the campaign descriptions and identified the best corresponding disease category for each term according to the groups of ICD-10-CM codes defined in the *Categorizing ICD-10-CM Codes* section. Each disease category was indicated as “present” or “absent” in the campaign. Interrater reliability was evaluated by using the Cohen κ. Discrepancies in labeling were reconciled in a group meeting among the students, and remaining disagreements were resolved by a senior physician. The presence or absence of each disease category was similarly determined by the algorithm, constituting a test set. Classification performance metrics for each disease category were then calculated in comparison with our expert consensus reference set. The reference set is provided in Multimedia Appendix 4. All analyses were done using Python version 3.8.8.

## Results

After applying the modifications described in the *Methods* section to CCSR, each ICD-10-CM code mapped to a single disease category. The NER and entity resolution models identified 6594 unique (276,020 total) ICD-10-CM codes among the 89,645 crowdfunding campaigns in our sample. Of the 6594 unique ICD-10-CM codes identified by the entity resolution model, 2884 (43.7%) did not have an exact match in the ICD-10-CM codes constituting our disease categories. Of these 2884 unmatched codes, 2544 (88.2%) were matched to a code with an identical stem and additional alphanumeric characters, indicating a more precise diagnosis. For example, the code “C5091” was identified by the NER model, and it represents cancer of the breast at an unspecified site. More precise codes, such as “C50911,” which indicates cancer of the right female breast, are included in the official CCSR documentation. Therefore, the iterative trimming process would match these codes and allow the unmatched code to inherit the proper disease category assignment.

Our manual review of the NER and entity resolution model outputs demonstrated the algorithm’s ability to appropriately identify and categorize misspelled diagnoses. For example, the phrase *brain aneruism* (correct spelling: *aneurysm*) was appropriately identified as a cerebral aneurysm and mapped to the *cardiovascular diseases* category. Another campaign contained the phrase *myeloid lukemia* (correct spelling: *leukemia*), but this was nonetheless appropriately categorized as a neoplasm.

Search terms for additional indicators of a disease category are shown in Table 1. Through our word search, we identified 3261 additional campaigns for which a medical condition was not otherwise detected with the NER model. Search terms for injuries and external causes allowed us to identify the most additional campaigns (n=1586), followed by search terms for cardiovascular diseases (n=598), neoplasms (n=486), genitourinary diseases (n=428), gastrointestinal diseases (n=135), and respiratory diseases (n=74). Furthermore, the word search often identified additional disease categories outside of those identified by the NER model. Among these campaigns, search terms identifying a new instance of neoplasms were most common (campaigns: n=19,079), followed by search terms for injuries and external causes (campaigns: n=9238), genitourinary diseases (campaigns: n=2086), cardiovascular diseases (campaigns: n=1919), gastrointestinal diseases (campaigns: n=648), and respiratory diseases (campaigns: n=440). The contribution of each individual search term is shown in Multimedia Appendix 5.

**Figure 2:**
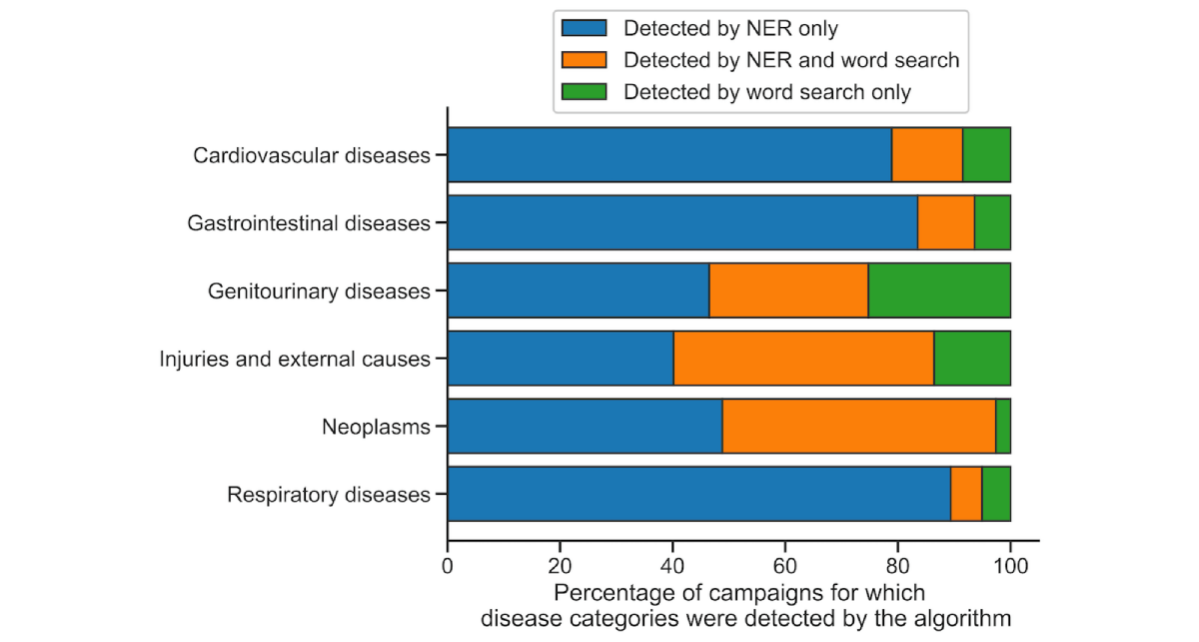
The relative contributions of the NER model and word search to detecting disease categories. All campaigns for which the disease categories on the y-axis were detected by the disease identification algorithm are presented. The colored bars represent the percentage of those campaigns for which the disease categories were detected by the NER model only (blue), the NER model and word search (orange), or the word search only (green). NER: named entity recognition.

The relative contribution of the word search to identifying disease categories that were not otherwise found by the NER model was small (Figure 2). Instances of disease categories that were detected exclusively via the word search ranged from 2.6% (993/38,221) for neoplasms to 25.2% (1185/4698) for genitourinary diseases. The word search more often identified disease categories that were also identified by the NER model. However, the exclusive contributions of the word search varied by disease category. For example, 94.9% (18,572/19,565) of the word search–identified campaigns mentioning neoplasms were identified by the NER model. Further, only 52.3% (269/514) of the word search–identified campaigns mentioning respiratory disease were identified by the NER model.

**Figure 3:**
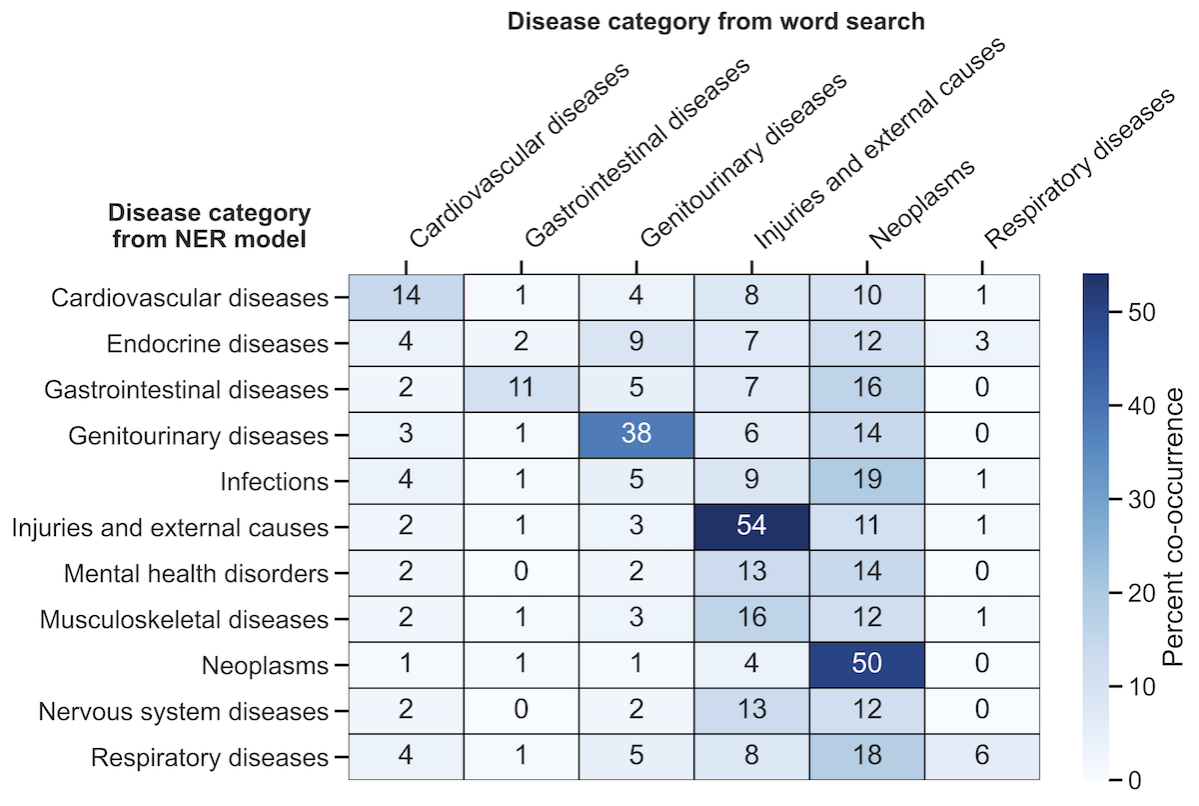
The co-occurrence of disease categories identified by the NER model and word search. The heat map values represent the percentage of campaigns containing the disease category in each row (identified by the NER model) that also contain the disease category in each column (identified via word search). NER: named entity recognition.

To understand the extent of overlap between disease categories that were identified by the word search and those that were identified by the NER model, we calculated how often the disease categories that were identified by each method co-occurred (Figure 3). The rates of co-occurrence also varied by disease category. For example, 53.5% (8371/15,634) of the NER model–identified campaigns mentioning injuries and external causes were also identified by the word search; the overlap was slightly lower for campaigns mentioning neoplasms (18,572/37,228, 49.9%) and genitourinary diseases (1329/3513, 37.8%). Co-occurrence rates were modest for the remaining disease categories that were common among those identified by the NER model and word search.

When preparing the reference set, the interrater reliability for detecting the presence of broad disease categories in the campaign descriptions was high (Cohen κ: range 0.69-0.96). The Cohen κ values for each disease category are shown in Multimedia Appendix 6. Discrepancies in coder annotation often occurred due to imprecise or vague descriptions of medical conditions. For example, one campaign described complications of a feeding tube, but it was unclear if the text sufficiently described a medical condition that was related to the gastrointestinal system. Other campaigns described a “sternum issue” or the patient getting “badly hurt” in an accident. After resolving these discrepancies, a reference set of disease categories in each campaign was established. The presence or absence of each disease category was then determined by the algorithm, and these outputs were compared to those in the reference set.

Classification performance metrics are detailed in Table 2 (additional values are included in Multimedia Appendix 7). The number of campaigns in our reference set that mentioned each disease category ranged from 18 (gastrointestinal diseases) to 162 (neoplasms). Classification performance also varied by disease category. When averaged across all disease categories and weighted by the number of campaigns that mentioned each disease category, the algorithm demonstrated an overall precision of 0.83 (range 0.48-0.97), a recall of 0.77 (range 0.42-0.98), an *F*_1_ score of 0.78 (range 0.56-0.96), and an accuracy of 95% (range 90%-98%). Representative examples of false positives and false negatives are provided in Multimedia Appendix 8.

**Table 1:** The keywords used to identify additional disease categories in campaign descriptions.

Disease category^a^	Keywords searched in campaign descriptions^b^	Representative examples from campaign descriptions
Injuries and external causes	*accident*, *injury/injuries/injured*, *crash*, *collision*, and *burn/burns/burned*	“[He] got into a serious accident in October. All four extremities were injured but the most severe were his legs.”
Cardiovascular diseases	*heart transplant* and *heart surgery*	“His cardiologist has informed him that a heart transplant is [his] only hope for survival.”
Neoplasms	*chemo/chemotherapy*, *radiation/radiotherapy*, and *bone marrow transplant*	“The chemotherapy did not stabilize the lymphoma so we were unable to move forward with the transplant.”
Genitourinary diseases	*dialysis* and *kidney/renal transplant*	“This disease resulted in my kidneys failing and having to start dialysis.”
Gastrointestinal diseases	*liver transplant*	“...the cirrhosis is incurable without a complete liver transplant.”
Respiratory diseases	*lung transplant*	“Her desire to live life...will only be possible with the double lung transplant.”

^a^Each disease category was indicated as present in a campaign if any of the corresponding terms were included in the campaign description.

^b^Keywords were selected during the exploratory reading of crowdfunding campaigns as indicators of a disease category that did not specify a diagnosis.

**Table 2:** Classification performance of the disease identification algorithm^a^.

Disease category	Campaigns in the reference set that mention disease category, n	Precision (95% CI)	Recall (95% CI)	*F*_1_ score	Accuracy (95% CI)
Cardiovascular diseases	82	0.92 (0.86-0.99)	0.74 (0.65-0.84)	0.82	0.94 (0.91-0.96)
Endocrine diseases	19	0.75 (0.54-0.96)	0.63 (0.41-0.85)	0.69	0.97 (0.96-0.99)
Gastrointestinal diseases	18	0.56 (0.33-0.79)	0.56 (0.33-0.79)	0.56	0.96 (0.94-0.98)
Genitourinary diseases	35	0.97 (0.90-1.03)	0.8 (0.67-0.93)	0.88	0.98 (0.97-0.99)
Infections	30	0.56 (0.41-0.71)	0.77 (0.62-0.92)	0.65	0.94 (0.91-0.96)
Injuries and external causes	53	0.69 (0.58-0.80)	0.92 (0.85-1.00)	0.79	0.94 (0.91-0.96)
Mental health disorders	20	0.48 (0.30-0.66)	0.7 (0.50-0.90)	0.57	0.95 (0.93-0.97)
Musculoskeletal diseases	45	0.64 (0.48-0.80)	0.51 (0.37-0.66)	0.57	0.91 (0.88-0.94)
Neoplasms	162	0.95 (0.91-0.98)	0.98 0.96-1.00)	0.96	0.97 (0.95-0.99)
Nervous system diseases	66	0.88 (0.76-0.99)	0.42 (0.31-0.54)	0.57	0.90 (0.86-0.93)
Respiratory diseases	29	0.92 (0.81-1.03)	0.76 (0.60-0.91)	0.83	0.98 (0.96-0.99)

^a^The average precision, recall, *F*_1_ score, and accuracy values are 0.83, 0.77, 0.78, and 0.95, respectively. Classification performance is based on a comparison to 400 campaigns that were annotated by a team of expert coders. The averages are weighted by the number of campaigns in the reference set that mention each disease category.

## Discussion

### Principal Results

We found that a disease identification algorithm using pretrained NER and entity resolution models linked to disease categories based on ICD-10-CM codes was able to detect 11 disease categories in crowdfunding campaigns with high precision and accuracy. Our analysis considered disease categories that represented a broad range of medical conditions. To our knowledge, this methodology is able to identify more disease categories in web-based medical crowdfunding campaigns than those identified by methods used in previous studies.

Our approach overcomes several limitations of previous work for identifying clinical populations in crowdfunding data. Crowdfunding campaigns contain many misspelled medical terms and informal synonyms (eg, *heart attack* vs *myocardial infarction*). Rule-based methods, such as keyword searches, require all acceptable terms for a given medical condition to be defined beforehand. Therefore, failing to account for alternative phrasings of medical conditions, which are expected in a large corpus, could significantly undermine the sensitivity of this approach. In contrast, NER models predict the probability of a certain word or phrase representing a medical condition and can account for variations in spelling and syntax. Another shortcoming of previous work is treating disease categories as mutually exclusive [6-9]. For example, one campaign may be exclusively categorized into the *neoplasms* category even if the campaign also mentions cardiovascular conditions. In our exploratory reading, we found that campaigns often mentioned multiple medical conditions across disease categories. To reflect the co-occurrence of disease categories, our approach treats disease categories as mutually inclusive. There is no external performance benchmark against which to evaluate our results, and to our knowledge, we are the first to report comprehensive evaluation metrics for a method that allows for multi-class disease category labeling and is scalable to medical crowdfunding text.

Based on our team’s exploratory reading of crowdfunding campaigns, we incorporated a word search alongside the NER model to identify additional disease categories that were not explicitly medical diagnoses. In general, the unique contribution of word search to the disease identification algorithm was modest. Although the word search identified additional campaigns for which the NER model did not detect any medical diagnoses, these campaigns represented a small proportion of the total campaigns in our sample. Furthermore, campaigns for which a disease category was found via the word search often had the same disease category detected by the NER model. Because we considered the presence or absence of disease categories at the level of entire campaigns, the word search results were often redundant to those from the NER model.

We examined how often a given disease category was detected in a campaign by both the NER model and word search. The co-occurrence rates corroborate the observation that multiple disease categories are often mentioned in the same campaign and underline the limitations of single-class disease category categorization, which has been used in previous work. In addition, while some disease categories (including genitourinary diseases, neoplasms, and injuries and external causes) were frequently found by both the NER model and word search in the same campaign, lower co-occurrence rates were observed among other disease categories. This may reflect the fact that our word search included a relatively narrow set of procedures or treatments when compared with those for the broad scope of medical diagnoses on which the NER model was trained. For example, it is not surprising that among the campaigns that were identified to mention cardiovascular diseases by the NER model, only 13.8% (1506/10,912) were found to contain mentions of heart surgery or a transplant.

A word search is, by definition, a rule-based approach and is therefore subject to the limitations discussed above. Although including a word search did enable the detection of additional campaigns and disease categories, it is fundamentally limited by the scope of included search terms. Therefore, while the search terms included in our algorithm were informed by an exploratory reading, future work should explore the use of the NER-based detection of procedures and treatments to capitalize on the flexibility of such methods for detecting additional clinical entities in patient-authored text.

Using pretrained NER and entity resolution models and disease categories based on ICD-10-CM codes provides a convenient and scalable method for structuring medical crowdfunding data. To our knowledge, there are no pretrained NER models that can detect a broad range of medical conditions in a corpus authored by members of the general public. Most medical NER models are trained on clinical documentation from electronic health records [18], though the particular NER model used in our study was trained on proprietary data [15]. Nevertheless, we found that one such NER model can be successfully applied to nonclinical texts, suggesting that similar approaches are likely to be effective across a much broader range of free text, such as social media posts.

### Limitations

Our study has several limitations. First, several disease categories were relatively infrequent in our reference set. This may have limited the classification performance for those disease categories (eg, gastrointestinal diseases). Second, the resolution of medical diagnoses to ICD-10-CM codes was often imperfect, resulting in clearly stated diagnoses sometimes being translated to an incorrect code. Third, an additional challenge with using ICD-10-CM codes was the lack of consistent formatting among the CCSR codes and the entity resolution model outputs. Codes without an exact match in corresponding data make it difficult to preserve diagnosis-level accuracy, but categorizing codes into broad disease categories largely avoids this problem. Fourth, we excluded several disease categories from our analysis, including conditions associated with pregnancy, ocular and otologic diseases, hematologic and immune disorders, and chromosomal abnormalities. Future work should focus on identifying these disease categories. Fifth, we were unable to distinguish between incidental mentions of medical conditions and those directly related to a beneficiary’s expenses. Sixth, we reported accuracy as a part of standard model evaluation metrics, but this should be interpreted with caution, given the class imbalance in the reference set.

### Conclusions

To address the challenges of identifying medical conditions in crowdfunding text, we leveraged pretrained NER and entity resolution models to predict the presence or absence of broad disease categories in medical crowdfunding campaign text. We evaluated the algorithm against a rigorously established reference set and provided transparent classification metrics. This algorithm precisely and accurately detects disease categories representing a broad range of pathologies and addresses key limitations of previous work.

## Appendix

Multimedia Appendix 1 Reassigned Clinical Classifications Software Refined categories.

Multimedia Appendix 2 Disease category assignment from diagnosis chapters.

Multimedia Appendix 3 Assignment of *International Classification of Diseases, 10th Revision, Clinical Modification* codes to disease categories.

Multimedia Appendix 4 Reference set indicating the presence (1) or absence (0) of each disease category in the corresponding campaign URL.

Multimedia Appendix 5 Results of word search for procedures and treatments. The left panel shows the number of campaigns identified by each search term on the y-axis for which no other disease category was found by the named entity recognition (NER) model (ie, found exclusively by word search). The right panel shows the number of campaigns identified by each search term on the y-axis among campaigns that also had at least 1 disease category found by the NER model.

Multimedia Appendix 6 Interrater reliability for detecting broad disease categories in campaign descriptions.

Multimedia Appendix 7 Classification concordance between the disease identification algorithm and the annotated reference set.

Multimedia Appendix 8 Examples of false-positive and false-negative disease category assignments by the disease identification algorithm.

## Abbreviations

CCSR: Clinical Classifications Software Refined
ICD-10-CM: *International Classification of Diseases, 10th Revision, Clinical Modification*
NER: named entity recognition
