# Supplementary material for: A Disease Identification Algorithm for Medical Crowdfunding Campaigns: Validation Study
Source: J Med Internet Res. 2022 Jun 21;24(6):e32867. doi: 10.2196/32867 (PMC9257615; doi:10.2196/32867)
Supplement: Multimedia Appendix 1 [file jmir_v24i6e32867_app1.pdf]

**Multimedia Appendix 1. Reassigned CCSR categories.**

| **CCSR clinical category** | **Original ICD-10-CM diagnosis chapter** | **Reassigned ICD-10-CM diagnosis chapter** |
|---|---|---|
| Cardiac and circulatory congenital anomalies | Congenital Malformations, Deformations and Chromosomal Abnormalities | Diseases of the Circulatory System |
| Transient cerebral ischemia | Diseases of the Nervous System | Diseases of the Circulatory System |
| Hepatitis | Certain Infectious and Parasitic Diseases | Diseases of the Digestive System |
| Digestive congenital anomalies | Congenital Malformations, Deformations and Chromosomal Abnormalities | Diseases of the Digestive System |
| Neonatal digestive and feeding disorders | Certain Conditions Originating in the Perinatal Period | Diseases of the Digestive System |
| Genitourinary congenital anomalies | Congenital Malformations, Deformations and Chromosomal Abnormalities | Diseases of the Genitourinary System |
| Perinatal infections | Certain Conditions Originating in the Perinatal Period | Certain Infectious and Parasitic Diseases |
| Musculoskeletal congenital conditions | Congenital Malformations, Deformations and Chromosomal Abnormalities | Diseases of the Musculoskeletal System and Connective Tissue |
| Nervous system congenital anomalies | Congenital Malformations, Deformations and Chromosomal Abnormalities | Diseases of the Nervous System |
| Neonatal cerebral disorders | Certain Conditions Originating in the Perinatal Period | Diseases of the Nervous System |
| Respiratory congenital malformations | Congenital Malformations, Deformations and Chromosomal Abnormalities | Diseases of the Respiratory System |
| Respiratory distress syndrome | Certain Conditions Originating in the Perinatal Period | Diseases of the Respiratory System |
| Respiratory perinatal condition | Certain Conditions Originating in the Perinatal Period | Diseases of the Respiratory System |
| Disorders of teeth and gingiva[a] | Diseases of the Digestive System | Other |

a. ICD-10-CM codes in this CCSR clinical category were not reassigned to a particular diagnosis chapter; rather, they flagged for removal with ICD-10-CM codes assigned to the “Other” disease category.
