# Supplementary material for: A Disease Identification Algorithm for Medical Crowdfunding Campaigns: Validation Study
Source: J Med Internet Res. 2022 Jun 21;24(6):e32867. doi: 10.2196/32867 (PMC9257615; doi:10.2196/32867)
Supplement: Multimedia Appendix 2 [file jmir_v24i6e32867_app2.pdf]

Multimedia Appendix 2. Disease category assignment from diagnosis chapters.

**ICD-10-CM diagnosis chapter**	**Disease category**[a]
Diseases of the Circulatory System	Cardiovascular diseases
Endocrine, Nutritional and Metabolic Diseases	Endocrine diseases
Diseases of the Digestive System	Gastrointestinal diseases
Diseases of the Genitourinary System	Genitourinary diseases
Certain Infectious and Parasitic Diseases	Infections
Injury, Poisoning and Certain Other Consequences of External Causes	Injuries and external causes
Mental, Behavioral and Neurodevelopmental Disorders	Mental health disorders
Diseases of the Musculoskeletal System and Connective Tissue	Musculoskeletal diseases
Neoplasms	Neoplasms
Diseases of the Nervous System	Nervous system diseases
Diseases of the Respiratory System	Respiratory diseases
Factors Influencing Health Status and Contact with Health Services	Other
Symptoms, Signs and Abnormal Clinical and Laboratory Findings, Not Elsewhere Classified	Other
Unacceptable principal diagnosis (inpatient data) or first-listed diagnosis (outpatient data)	Other
Diseases of the Blood and Blood Forming Organs and Certain Disorders Involving the Immune Mechanism	Other
Diseases of the Ear and Mastoid Process	Other
Diseases of the Eye and Adnexa	Other
Congenital Malformations, Deformations and Chromosomal Abnormalities	Other
Diseases of the Skin and Subcutaneous Tissue	Other
Certain Conditions Originating in the Perinatal Period	Other
Pregnancy, Childbirth and the Puerperium	Other

a. ICD-10-CM diagnosis chapters were renamed to distinguish the reclassified disease categories from the official ICD-10-CM and CCSR data.
