# Supplementary material for: A Disease Identification Algorithm for Medical Crowdfunding Campaigns: Validation Study
Source: J Med Internet Res. 2022 Jun 21;24(6):e32867. doi: 10.2196/32867 (PMC9257615; doi:10.2196/32867)
Supplement: Multimedia Appendix 5 [file jmir_v24i6e32867_app5.pdf]

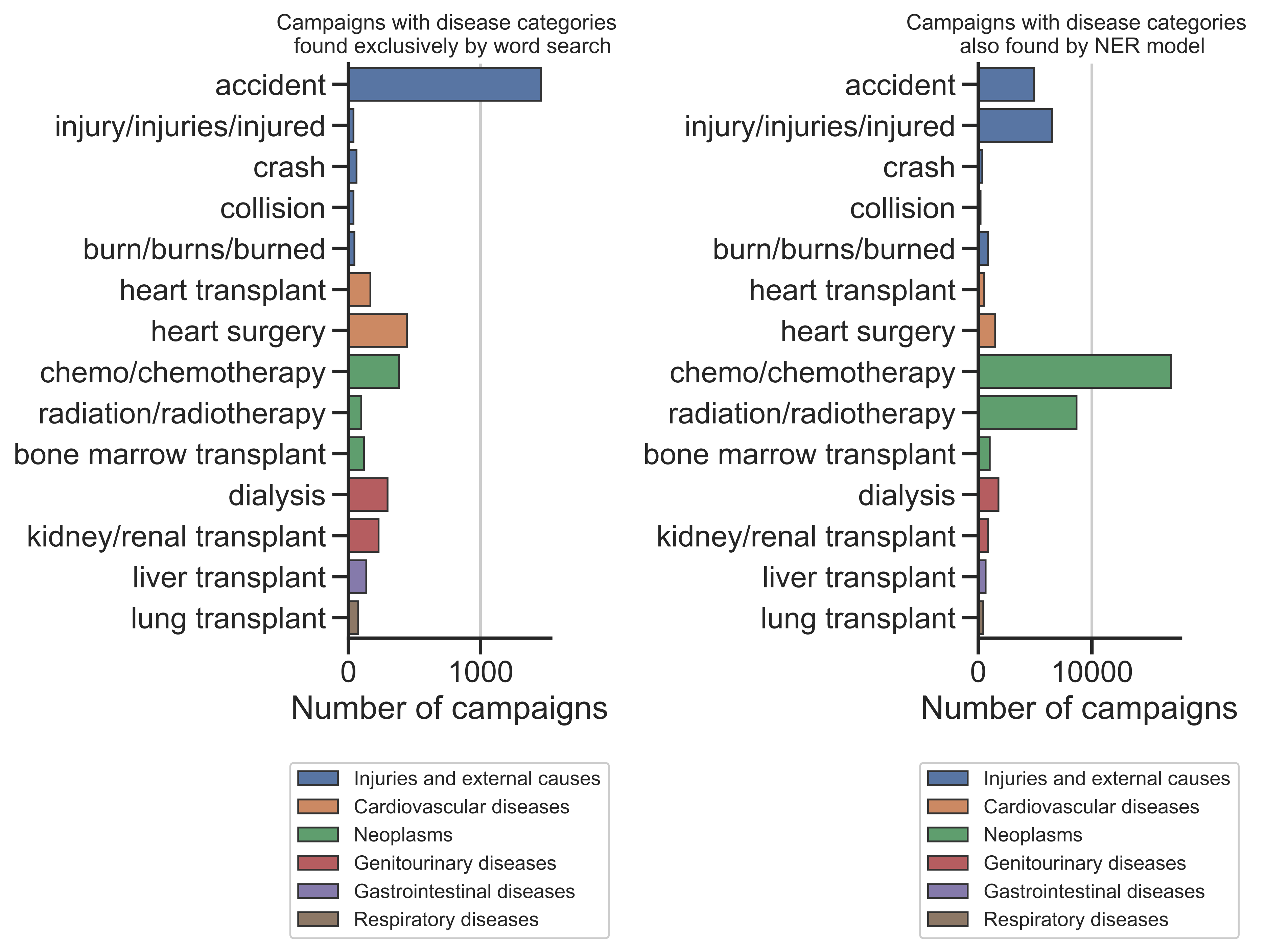

Campaigns with disease categories found exclusively by word search
Campaigns with disease categories also found by NER model
accident
injury/injuries/injured
crash
collision
burn/burns/burned
heart transplant
heart surgery
chemo/chemotherapy
radiation/radiotherapy
bone marrow transplant
dialysis
kidney/renal transplant
liver transplant
lung transplant
0
1000
10000
Number of campaigns
Injuries and external causes
Cardiovascular diseases
Neoplasms
Genitourinary diseases
Gastrointestinal diseases
Respiratory diseases
