# Supplementary material for: A Disease Identification Algorithm for Medical Crowdfunding Campaigns: Validation Study
Source: J Med Internet Res. 2022 Jun 21;24(6):e32867. doi: 10.2196/32867 (PMC9257615; doi:10.2196/32867)
Supplement: Multimedia Appendix 6 [file jmir_v24i6e32867_app6.pdf]

Multimedia Appendix 6. Inter-rater reliability for detecting broad disease categories in campaign description.

**Disease category**	**Cohen's kappa**
Neoplasms	0.96
Infections	0.93
Endocrine diseases	0.91
Injuries and external causes	0.89
Cardiovascular diseases	0.88
Genitourinary diseases	0.85
Musculoskeletal diseases	0.85
Respiratory diseases	0.85
Mental health disorders	0.84
Nervous system diseases	0.76
Gastrointestinal diseases	0.69
