# Supplementary material for: A Disease Identification Algorithm for Medical Crowdfunding Campaigns: Validation Study
Source: J Med Internet Res. 2022 Jun 21;24(6):e32867. doi: 10.2196/32867 (PMC9257615; doi:10.2196/32867)
Supplement: Multimedia Appendix 7 [file jmir_v24i6e32867_app7.pdf]

Multimedia Appendix 7. Classification concordance between disease identification algorithm and annotated reference set.

Disease category	True positives	False positives	True negatives	False negatives
Cardiovascular diseases	61	5	313	21
Endocrine diseases	12	4	377	7
Gastrointestinal diseases	10	8	374	8
Genitourinary diseases	28	1	364	7
Infections	23	18	352	7
Injuries and external causes	49	22	325	4
Mental health disorders	14	15	365	6
Musculoskeletal diseases	23	13	342	22
Neoplasms	159	9	229	3
Nervous system diseases	28	4	330	38
Respiratory diseases	22	2	369	7
