# Supplementary material for: A Disease Identification Algorithm for Medical Crowdfunding Campaigns: Validation Study
Source: J Med Internet Res. 2022 Jun 21;24(6):e32867. doi: 10.2196/32867 (PMC9257615; doi:10.2196/32867)
Supplement: Multimedia Appendix 8 [file jmir_v24i6e32867_app8.pdf]

Multimedia Appendix 8. Examples of false positive and false negative disease category assignments by the disease identification algorithm**.**

Disease category	Error	Excerpt from campaign description	Text supporting reference set annotation	Text identified by algorithm[a]
Cardiovascular diseases	False positive	“[They] are some of the nicest and most genuine people I have ever met and to watch them go through this is heart breaking.”	N/A	“heart breaking”
Cardiovascular diseases	False negative	“[He] had an aortic dissection about a week ago.”	“aortic dissection”	N/A
Endocrine diseases	False positive	“The ONLY time I have seen my daughter improve is when we treated the underlying infection and nutritional deficiencies through healthy diet and supplements.”	N/A	“nutritional deficiencies”
Endocrine diseases	False negative	“[She] originally went to the Cleveland Clinic for surgery to remove her thyroid after a diagnosis with Graves' disease.”	“Grave’s disease”	N/A
Gastrointestinal diseases	False positive	“I’m at [the] hospital with bacterial endocarditis, MRSA in my bloodstream, septic emboli, two heart valves with vegetation(clot) buildup on them, infection pockets in both lungs”	N/A	“infection pockets”
Gastrointestinal diseases	False negative	“While she was there, she had multiple emergency surgeries stemming from an ulcer.”	“ulcer”	N/A
Genitourinary diseases	False positive	“by working with those who are working to change how people are treated with psychiatric SYMPTOMS by looking at the underlying medical cause and what is wrong with the PHYSICAL body such as candida and food allergies.”	N/A	“candida”
Genitourinary diseases	False negative	“An infection broke out and her kidneys are unable to fight off the infections as needed, and are slowly shutting down.”	“kidneys...are slowly shutting down”	N/A
Infections	False positive	“My sickness continued into adulthood, my weight became a deadly 555 lbs I was super obese.”	N/A	“sickness”
Infections	False negative	“He also developed MRSA and pneumonia due to intubation.”	“MRSA”	N/A
Injuries and external causes	False positive	“The disease is caused from a tick bite that goes untreated.”	N/A	“tick bite”
Injuries and external causes	False negative	“I broke two teeth and was having severe pain in an already broken tooth.”	“broken tooth”	N/A

Mental health disorders	False positive	"Shannon Heika is a beautiful 22 year old who was just diagnosed with Blastic plasmacytoid dendritic cell neoplasm (BPDCN) and now she's in a fight for her life."	N/A	"BPDCN"
Mental health disorders	False negative	"I've been struggling with Bipolar Type 1 for over a year now."	"Bipolar Type 1"	N/A
Musculoskeletal diseases	False positive	"He sustained many injuries including crushed veterbrae and a shattered pelvis."	NA	"shattered pelvis"
Musculoskeletal diseases	False negative	"I fell down stairs at work in 2005 and have been recovering from a Traumatic Brain Injury and chronic neck pain since then."	"chronic neck pain"	N/A
Neoplasms	False positive	"This type of surgical treatment removes the endometriosis from below its base roots embedded into the organ (instead of just burning the surface via ablation) and effectively slows down progression of this cancer-like disease."	N/A	"cancer-like disease"
Neoplasms	False negative	"The results were that she had Acute Leukemia and that she would need to be transported to another hospital"	"acute leukemia"	N/A
Nervous system diseases	False positive	"...now my mother was diagnosed with Dementia in Germany"	N/A	"dementia"
Nervous system diseases	False negative	"Back in July, [he] was having terrible headaches and nausea."	"headaches"	N/A
Respiratory diseases	False positive	"He belives [sic] my mother only has Pulmonary Sarcodosis [sic]."	N/A	"pulmonary sarcodosis [sic]"
Respiratory diseases	False negative	"As of right now she has a collapsed lung, fractured skull in two places, face fracture on her right side, air on her brain and belly"	"collapsed lung"	N/A

a. Only includes segments of text identified for the row's corresponding disease category. Text corresponding to other disease categories may be present in the excerpt from the campaign description.

N/A: not applicable
